# Development of a Practical Synthesis of the **8-FDC** Fragment of OPC-167832

**DOI:** 10.1021/acsomega.1c06996

**Published:** 2022-02-18

**Authors:** Vijayagopal Gopalsamuthiram, Dang Binh Ho, Cheryl L. Peck, Vasudevan Natarajan, Toolika Agrawal, Justina M. Burns, John Bachert, Daniel W. Cook, Rodger W. Stringham, Ryan Nelson, Saeed Ahmad, B. Frank Gupton, David R. Snead, D. Tyler McQuade, Rajappa Vaidyanathan, Kai Donsbach, Joshua D. Sieber

**Affiliations:** † Medicines for All Institute, Virginia Commonwealth University School of Engineering, Chemical Development 737 N 5th Street, Richmond, Virginia 23298-0100, United States; ‡ Department of Chemistry, Virginia Commonwealth University, 1001 West Main Street, Richmond, Virginia 23284-3208, United States

## Abstract

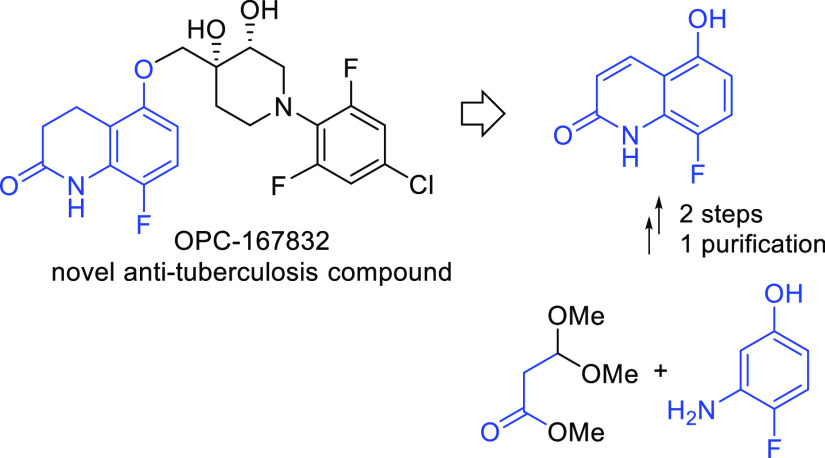

A concise and practical synthesis has been developed to provide the 8-fluoro-5-hydroxy-3,4-diydrocarbostyril (**8-FDC**) fragment of OPC-167832 in 41% yield and in >99% purity over four steps from 3-amino-4-fluorophenol. The key feature of this process is the development of a telescoped one-pot synthesis of the quinolone via a chemoselective amidation/acid-induced cyclization that allows for simple product isolation without the need for column chromatography.

## Introduction

**Figure 1:**
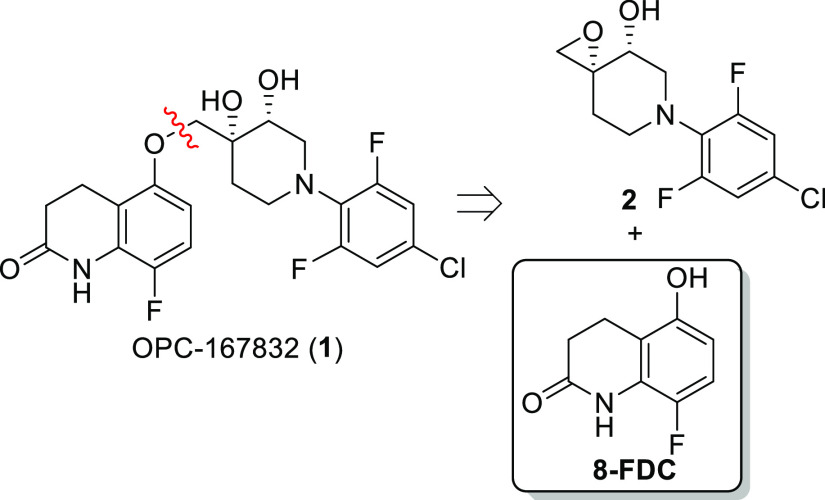
OPC-167832.

Tuberculosis (TB) is a contagious bacterium infection caused by *Mycobacterium tuberculosis* (mtb) and was still the leading cause of death worldwide by infectious disease in 2019.^1^ In 2017, 10 million people were infected by TB, and 1.6 million deaths by TB occurred including 230,000 children.^2^ Treatments for TB are available; however, drug resistance to these treatments is an ongoing problem.^1,3^ First-line treatments were discovered as early as 1952. Drug-resistant strains of TB include multidrug-resistant TB (MDR-TB) and extensively drug-resistant TB (XDR-TB). The only current compounds available to treat MDR-TB are delamanid, pretomanid, and bedaquiline in combination with other TB drugs.^1,3^ However, identification of novel compounds with unique mechanisms of action is needed to combat drug resistance and develop shorter less toxic treatment regimens. In this regard, OPC-167832^4^ (**1**, Figure 1), developed by Otsuka Pharmaceutical Co., Ltd., is a promising compound for the treatment of MDR-TB strains in combination therapy that operates by a unique mode of action^1^ and has received fast track status for development by the US FDA.^5^

**Scheme 1:**
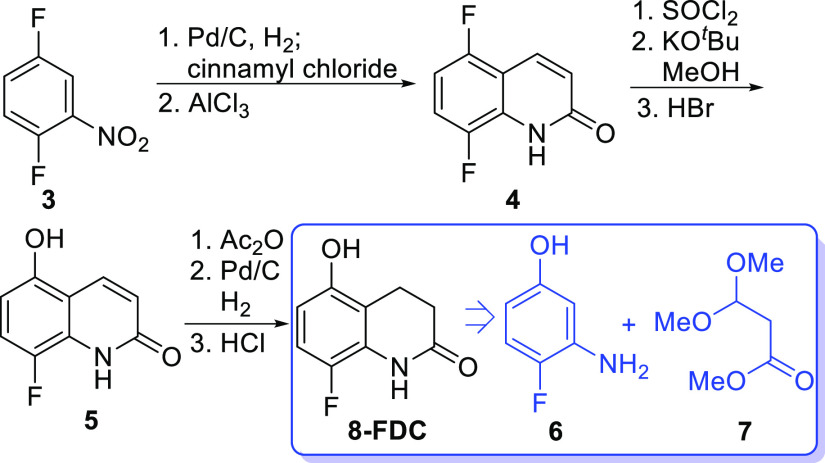
Reported^4b^ and Proposed Synthetic Strategy to **8-FDC**

Due to the biological significance of **1**,^1,4^ efficient synthetic access to this compound is important to enable supply. The current reported synthesis of **1**(4b,4c) utilizes a final coupling of two fragments (**2** and **8-FDC**, Figure 1). In collaboration with Otsuka Pharmaceutical Co., Ltd., our group became interested in investigating improved synthetic routes to one of the key coupling fragments, **8-FDC**. Access to **8-FDC** suffers from a long synthetic sequence (nine chemical steps and eight “reaction pots”) starting from fluorinated nitrobenzene **3** (Scheme 1).^4b^ This approach mainly suffers from multiple functional group interconversion steps, including replacement of the 5-fluoro group of **3** with the requisite OH-group of **8-FDC**. This F to O swap (**4** → **5**) leads to the incorporation of several additional steps into the synthetic route. As a result, we decided to investigate an alternative synthetic design from aniline **6**(6) with the requisite 5-OH group already present in the reaction with methyl 3,3-dimethoxypropionate (**7**) to access the desired pyridone scaffold of **8-FDC**. Importantly, both **6** and **7** are commercially available, and synthetic procedures to access **6** on up to 5 kg scale have been reported.^7^ Herein, we describe the successful synthesis of **8-FDC** utilizing this approach.^8^

## Results and Discussion

**Scheme 2:**
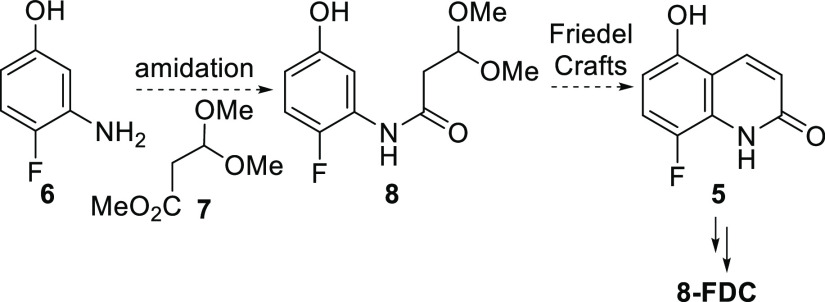
Planned Forward Synthesis of **8-FDC**

To realize our proposed synthesis plan in Scheme 1, the envisioned forward synthesis is given in Scheme 2. Chemoselective amide formation between **6** and **7** was desired to provide **8** that may be converted to quinolone **5** by a Friedel–Crafts type process. Recently, this approach for the synthesis of quinolones was reported.^9^ However, we were unsuccessful in identifying chemoselective conditions for formation of amide **8** over the ester formed from reaction of **7** with the phenol group of **6**. As a result, we next investigated the synthesis of amide **8** from the coupling of acid **10** prepared from the hydrolysis of **7** with aqueous NaOH (Table 1). Of the various carboxylic acid activating agents studied, MsCl and PivCl appeared to be the most promising for the formation of product **8** (entries 1–4). A subsequent survey of bases and solvents utilizing PivCl as the activating agent was then carried out (entries 4–15). Among the bases analyzed, DBU (1,8-diazabicyclo(5.4.0)undec-7-ene) and Hunig’s base (DIPEA, diisopropylethylamine) afforded the highest amounts of the desired amide **8** (entries 8 and 9), but the latter was preferred for its lower cost. With regard to the reaction solvent, toluene provided the maximum conversion to **6** and also accounted for the highest yield (entry 13).

**Table 1:**
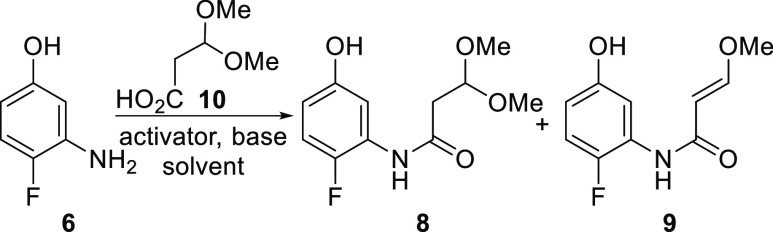
Optimization of Chemoselective Amidation of **10** with Aniline **6**^a^

entry	solvent	activator	base	% **6**^b^	% **8**^b^^,^^c^	% **9**^b^
1	MeCN	MsCl	Et_3_N	20	53 (39)	1.5
2	MeCN	*p*-TsCl	Et_3_N	3.9	35 (30)	1.4
3	MeCN	ClCO_2_^*i*^Pr	Et_3_N	37	36	13
4	MeCN	PivCl	Et_3_N	13	78 (64)	0.9
5	MeCN	PivCl	K_2_CO_3_	41	16	25
6	MeCN	PivCl	NaHCO_3_	13	5.1	60
7	MeCN	PivCl	pyridine	8.6	68 (61)	7.6
8	MeCN	PivCl	*i*Pr_2_NEt	12	75 (66)	0.7
9	MeCN	PivCl	DBU	5.6	80 (86)	1.4
10	THF	PivCl	Et_3_N	2.1	80	7.7
11	THF	PivCl	*i*Pr_2_NEt	3.6	82 (83)	4.0
12	C_6_H_5_Cl	PivCl	*i*Pr_2_NEt	0	67	10.1
13	PhMe	PivCl	*i*Pr_2_NEt	0.1	95 (92)	0.4
14	CPME	PivCl	*i*Pr_2_NEt	0.5	36	20
15	DCM	PivCl	*i*Pr_2_NEt	0	97 (89)	0.1

a Conditions: 0.93–1.55 mmol **10**, base (1.25 equiv), and activator (1.25 equiv) from 0 °C to rt for 3 h and then 0.93–1.55 mmol **6**, rt, 16 h.

b Value represents the area % at 210 nm by reverse phase HPLC analysis after 16 h.

c Value in parentheses represents assay yield, as determined by quantitative ^1^H NMR analysis of the unpurified reaction mixture using mesitylene as the analytical standard.

**Figure 2:**
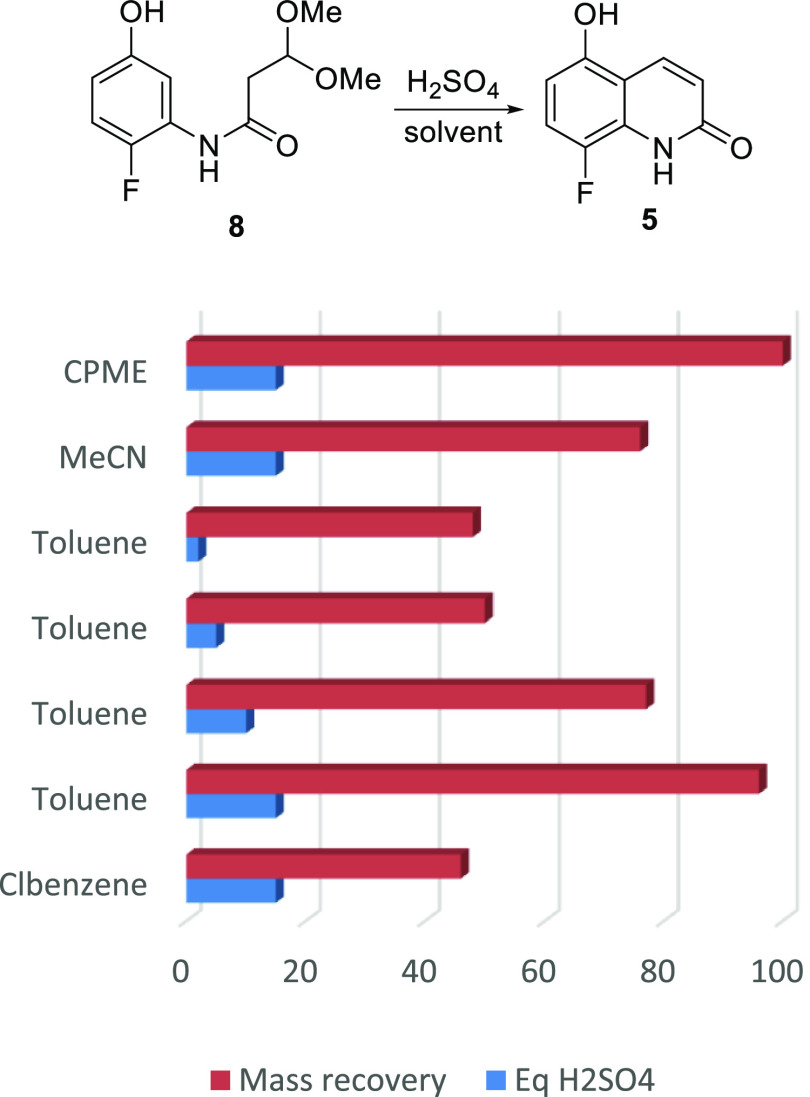
Mass recovery vs sulfuric acid loading and solvent.

With the intent to telescope the overall synthesis, a solvent study for the cyclization of amide **8** to the quinolone **5** was next performed. A quick comparative analysis of the efficiency of the reaction in various solvents was made on the basis of the amount of the solid mass isolated post precipitation from the reaction mixture by pouring into ice cold water, and the results are captured in Figure 2.

**Scheme 3:**
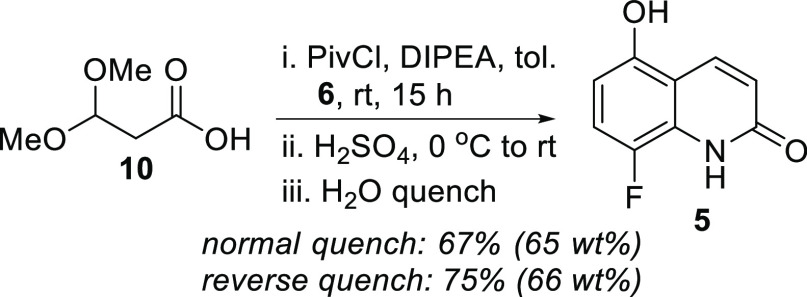
Telescoped One-Pot Quinolone Synthesis

Although cyclopentyl methyl ether (CPME) and toluene gave excellent mass recovery, toluene was preferred for telescoping the reaction due to its better performance over CPME in the amidation step. In toluene, 67% assay yield of the quinolone was obtained in 75 wt % purity by quantitative ^1^H NMR analysis after direct precipitation from the reaction mixture using water (normal quench). Telescoping the process into a one-pot synthesis of quinolone **5** was found to be successful (Scheme 3), and the quinolone was isolated in 65 wt % purity (quantitative ^1^H NMR analysis) after precipitation. Due to the significant exotherm observed during the normal quenching procedure, a reverse quench by transferring the reaction mixture to ice cold water was next tested in an effort to increase the reaction yield. Gratifyingly, this led to an improved yield of 75% on a 10 g scale.

**Figure 3:**
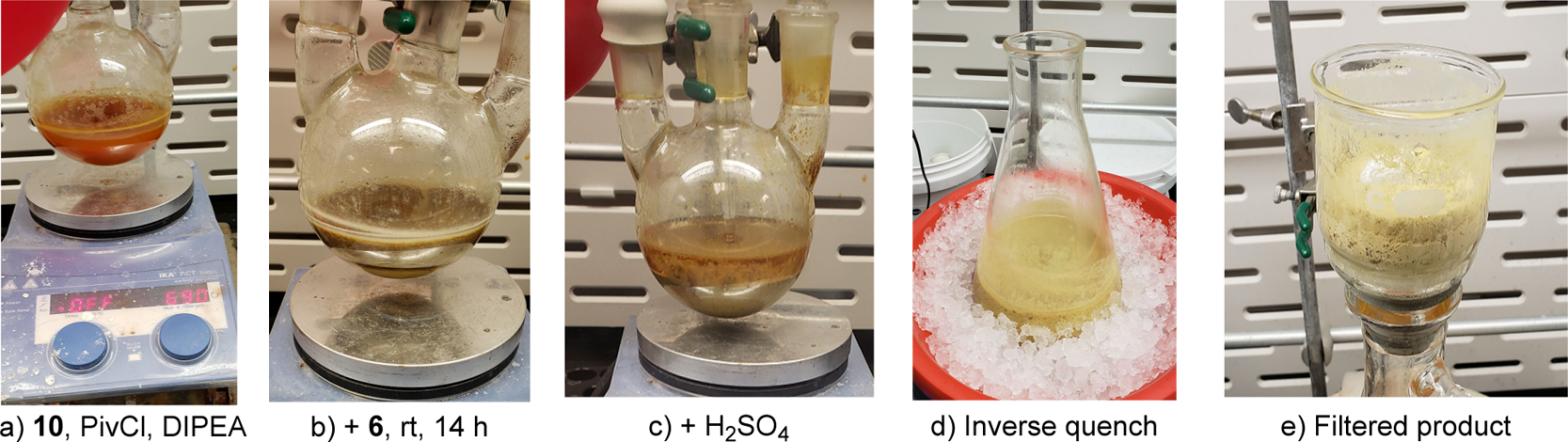
Reaction progress at different stages utilizing reverse-quenching. (a) Reaction of **10**, PivCl, and DIPEA. (b) After addition of **6**, rt, 14 h. (c) After addition of H_2_SO_4_. (d) After inverse quench. (e) Filtered product.

The stages of this one-pot sequence are shown in Figure 3. The initial reaction solution of acid **10** in toluene was homogeneous, however, upon addition of the pivaloyl chloride, the reaction becomes biphasic. After addition of DIPEA, the reaction remained biphasic and resulted in the formation of precipitated DIPEA·HCL and amide **8** (solubility of **8** in toluene is ∼0.3 mg/mL) after overnight agitation. H_2_SO_4_ was then slowly added using the addition funnel at 0 °C to control the exotherm, leading to the formation of a triphasic reaction mixture. Final reverse-quenching into water induces the precipitation of quinolone **5** that was isolated by filtration. Losses of **5** to the filtrate were determined to be 348 mg in 390 mL (∼3%) when using a normal quench mode and 656 mg in 448 mL (∼6%) when a reverse quench was employed, as determined by quantitative reverse phase HPLC analysis.

Purification of the resultant quinolone obtained from direct precipitation with water can be achieved using either recrystallization from 80:20 MeOH/H_2_O or by treatment with 10 V aqueous sodium bicarbonate solution. Using these procedures, **5** could be obtained in 100 wt % purity with 78% recovery after recrystallization with MeOH/H_2_O or in 93 wt % purity in 88% recovery if treated with aqueous NaHCO_3_. As a result, the overall yield of **5** from acid **10** was 59% when recrystallization was employed or 62% when utilizing a NaHCO_3_ reslurry.

**Scheme 4:**
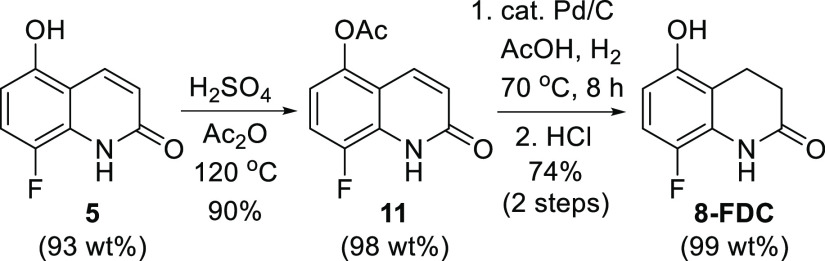
Conversion of Quinolone **7** to **8-FDC**

Since an increased overall yield of **5** was obtained when purifying the material from aqueous NaHCO_3_, this quality material was attempted to be converted to **8-FDC** by the established literature procedure^4b,4c^ to ascertain whether 93 wt % material was acceptable (Scheme 4). Gratifyingly, **5** was smoothly converted to **8-FDC** in comparable yields without any issue.

In conclusion, we have developed a concise process using cheap reagents and starting materials accessible on a bulk scale for the preparation of the key dihydroquinolone (**8-FDC**) fragment of OPC-167832. The novel process described herein features a telescoped one-pot operation for the preparation of quinolone **5** from 3-amino-4-fluorophenol and 3,3-dimethoxy propionic acid without the need for isolation of any of the intermediates. The method described herein should, in principle, be broadly applicable for selective acylation of a wide variety of aminophenols and would therefore be significant for efficient synthesis of various biologically active molecules.

## Experimental Section

### General

All reactions were carried out under a nitrogen atmosphere unless otherwise indicated. Glassware was predried in an oven prior to use. 3-Amino-4-fluorophenol was purchased from Oakwood, methyl 3,3-dimethoxy propionate was purchased from TCI chemicals, acetic anhydride was purchased from Chem Impex, and trimethylacetyl chloride and 10% Pd/C and *N*,*N*-diisopropylethylamine were purchased from Sigma-Aldrich. Toluene and methanol of reagent grade were purchased from J. T. Baker, whereas methyl *tert*-butyl ether (MTBE), NaOH (pellets), dimethyl fumarate, mesitylene, hydrochloric acid, sulfuric acid, and acetic acid were purchased from Sigma-Aldrich.

#### 3,3-Dimethoxpropanoic Acid (**10**)

To a 100 mL round-bottom flask with a stir bar is charged 20 mL of water followed by 8.1 mL of 10 M NaOH (30%). Ester **7** (10 g) was charged and stirred at 55 °C for 2 h. TLC (30% EtOAc/hex) and ^1^H NMR spectroscopy of a worked-up aliquot [obtained by quenching 20 μL of solution with a few drops of concentrated HCl until pH was acidic, followed by extraction with methyl *tert*-butyl ether (MTBE, 2 mL) and concentration] showed consumption of the starting ester. The reaction mixture was then cooled to room temperature and washed with MTBE (1 × 30 mL). To the aqueous layer was then added 6.7 mL of concentrated HCl over ∼10 min keeping the internal temperature below 18 °C using an ice bath. The batch was warmed up to room temperature, and 4.5 g of sodium chloride was added. The reaction mixture was stirred for about 10 min to dissolve the salt, and the organic layer was extracted with MTBE (3 × 50 mL). The organics were further dried with Na_2_SO_4_, filtered, and concentrated in vacuo to provide 9.72 g (99%) of **10** as a near colorless oil in 91.7 wt % purity, as determined by quantitative ^1^H NMR spectroscopic analysis using dimethyl fumarate as the analytical standard. Spectral data were identical to the literature.^10^

#### 8-Fluoro-5-hydroxyquinolin-2(1*H*)-one (**5**)

3,3-Dimethoxy propionic acid **10** (9.5 g, 63 mmol, 89 wt %) was weighed into a three neck round-bottom flask having mechanical stirring and under an atmosphere of N_2_. Reagent grade toluene (95 mL) was added followed by *N*,*N*-diisopropyl ethylamine (14 mL, 1.25 equiv, 79 mmol) at room temperature. The reaction mixture was then cooled to 0 °C, and trimethylacetyl chloride (9.7 mL, 1.25 equiv, 79 mmol) was added dropwise over 5 min. The reaction mixture became cloudy post addition with the formation of a precipitate. The reaction was then warmed to room temperature and stirred for 4 h. 3-Amino-4-fluorophenol (**6**, 8.9 g, 63 mmol, 1 equiv, 90 wt %) was added neatly as a solid to the reaction mixture at room temperature. The heterogeneous biphasic reaction mixture was then allowed to stir for overnight. HPLC analysis post overnight showed clean formation of **8**. At this stage, the reaction was again cooled to 0 °C, and H_2_SO_4_ (50 mL, 15 equiv, 950 mmol) was added via an addition funnel dropwise over 45 min. Post addition, the reaction was warmed up again to room temperature and stirred for 45 min. At this stage, clear formation of two layers was observed. The reaction mixture was transferred carefully into cold water (∼30 V, 300 mL) in a 500 mL Erlenmeyer flask kept in an ice bath. To the viscous and oily nature of the bottom sulfuric acid layer that still remained in the round-bottom flask was added additional water (10 V, 100 mL) successively in portions dropwise in cold condition, and the precipitate obtained was combined with the material in the Erlenmeyer flask mentioned above. After stirring the contents for 30 min at room temperature, the obtained precipitate was filtered and successively washed with water and toluene (5 V each). Lastly, the precipitate was washed with MTBE (5 V) and dried for 30 min under house vacuum. The Büchner funnel was kept for drying inside a beaker in a vacuum oven at 60 °C for 15 h to provide 12.8 g (75%) of **7** as a light-yellow solid in 66 wt % purity, as determined by quantitative ^1^H NMR spectroscopic analysis using mesitylene as the analytical standard. This crude material was added to 130 mL, 10 V of 9% aqueous sodium bicarbonate solution and stirred at room temperature for 1 h. After gas evolution subsided (∼40 min), the solid was collected by filtration, washed with water (2 × 20 mL), and dried in a vacuum oven at 60 °C for 15 h under a gentle stream of N_2_ to afford 7.5 g (62% overall, 82% recovery) of **5** as a beige colored solid in 93 wt % purity, as determined by quantitative ^1^H NMR spectroscopic analysis using mesitylene as the analytical standard. Spectral data were consistent with the literature.^4b,4c^^1^H NMR (DMSO-*d*_6_, 600 MHz): δ 11.6 (br s, 1H), 10.3 (br s, 1H), 8.01 (d, 1H, *J* = 12 Hz), 7.19 (t, 1H, *J* = 12 Hz), 6.51 (dd, *J* = 12, 6 Hz), 6.46 (d, 1H, *J* = 12 Hz). ^13^C{^1^H} NMR (151 MHz, DMSO-*d*_6_): δ 162.0, 150.4, 143.0, 141.4, 135.0, 128.2, 120.9, 116.3, 110.2, 100.6.

#### Analytical Data for **8**

^1^H NMR (MeOH-*d*_4_, 600 MHz): δ 7.43 (dd, 1H, *J* = 2.4, 6 Hz), 6.88 (t, 1H, *J* = 10 Hz), 6.42–6.50 (m, 1H), 4.77 (t, 1H, *J* = 6 Hz), 3.35 (s, 6H), 2.68 (d, 2H, *J* = 6 Hz). ^13^C{^1^H} NMR (151 MHz, MeOH-*d*_4_): δ 169.0, 153.2, 148.4, 146.8, 125.9, 114.9, 111.0, 110.0, 100.2, 53.0, 40.6. HRMS (ESI): *m*/*z* calcd for C_11_H_14_FNNaO_4_ [M + Na]^+^, 266.0805; found, [M + Na]^+^, 266.0789.

#### Synthesis of 8-Fluoro-5-hydroxy-3,4-diydrocarbostyril (8-Fluoro-5-hydroxy-3,4-dihydroquinolin-2(1*H*)-one, **8-FDC**)

To a suspension of **5** (2.00 g, 10.4 mmol, 93 wt %) in a 20 mL reaction vial was added 12 mL of acetic anhydride and heated to 120 °C for 2 h. The reaction mixture was cooled to room temperature (upon cooling, precipitation occurred) and poured into 10 V of iced water. The reaction mixture was stirred at this temperature for 1 h. The precipitate was collected and washed with 3 V of water to afford **11** as a beige colored solid (2.1 g, 90% yield, 98 wt %). The analytical data were identical in all respects to the literature.^4b,4c^

In a mini autoclave vessel, 10% Pd/C (20 mg, 10 wt % as a dry powder) was added to a suspension of **11** (0.200 g, 0.832 mmol) in 10 V of AcOH. The vessel was backfilled and vented with nitrogen followed by H_2_ at 60 psi (4 atm). The reaction mixture was then heated at 75 °C for 8 h. The vessel was then cooled to 40 °C and the autoclave was vented/replaced with N_2_. The residue was filtered through 2 wt % celite. The filtrate was evaporated to a white solid under reduced pressure to provide 214 mg (97% yield, 84 wt %) of acetate-protected **8-FDC** as a white solid. To a suspension of this material (214 mg) in 1 mL, 5 V of MeOH was added to concentrated HCl (5 V) in 1 mL. The reaction mixture was heated at 100 °C for 1 h. It was then cooled to 40 °C, and water (2 mL) was added (precipitation occurred) followed by stirring at 30 °C for 1 h. The reaction mixture was then cooled to 0 °C and stirred for an additional 1 h. The precipitate was then filtered and dried to provide 113 mg (77%, 75% over two steps) of **8-FDC** as a white solid in 99.3 wt % purity by quantitative ^1^H NMR spectroscopic analysis using mesitylene as the analytical standard. Analytical data of **8-FDC** exactly matched the original literature.^4b,4c^

## Supporting Information Available

Copies of ^1^H and ^13^C NMR spectra (PDF)
