# Supplementary material for: Development of a Practical Synthesis of the 8-FDC Fragment of OPC-167832
Source: ACS Omega. 2022 Feb 18;7(8):7223–8. doi: 10.1021/acsomega.1c06996 (PMC8892917; doi:10.1021/acsomega.1c06996)

Supporting Information

Development of a Practical Synthesis of the 8-FDC Fragment of OPC-167832

Vijayagopal Gopalsamuthiram,[a] Dang Binh Ho,[a,b] Cheryl L. Peck,[a] Vasudevan Natarajan,[a] Toolika Agrawal,[a,b] Justina M. Burns,[a] John Bachert,[a] Daniel W. Cook,[a] Rodger W. Stringham,[a] Ryan Nelson,[a] Saeed Ahmad,[a] B. Frank Gupton,[a] David R. Snead,[a] D. Tyler McQuade,[a] Rajappa Vaidyanathan,[a] Kai Donsbach,[a] and Joshua D. Sieber*,[a,b]

[a]*Chemical Development, Medicines for All Institute, 737 N 5th Street, Richmond, VA 23298-0100, USA.*

[b]*Department of Chemistry, Virginia Commonwealth University, 1001 West Main Street, Richmond, VA 23284-3208, USA.*

*Correspondence to: jdsieber@vcu.edu

Table of contents:

^{1}H and ^{13}C NMR data …………………………………………………………………………………………………… S2-S5

^{1}HNMR (600 MHz, $CDCl_3$):

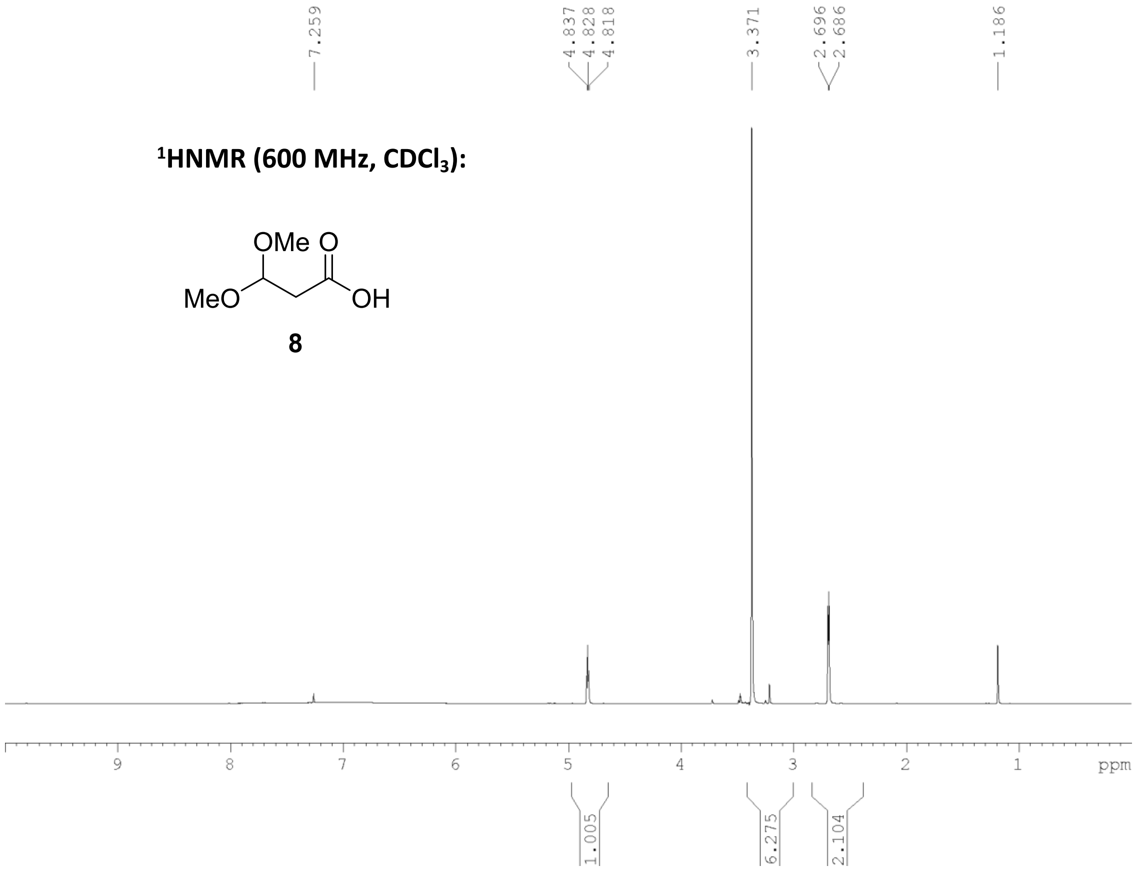

^{13}CNMR (151 MHz, $CDCl_3$):

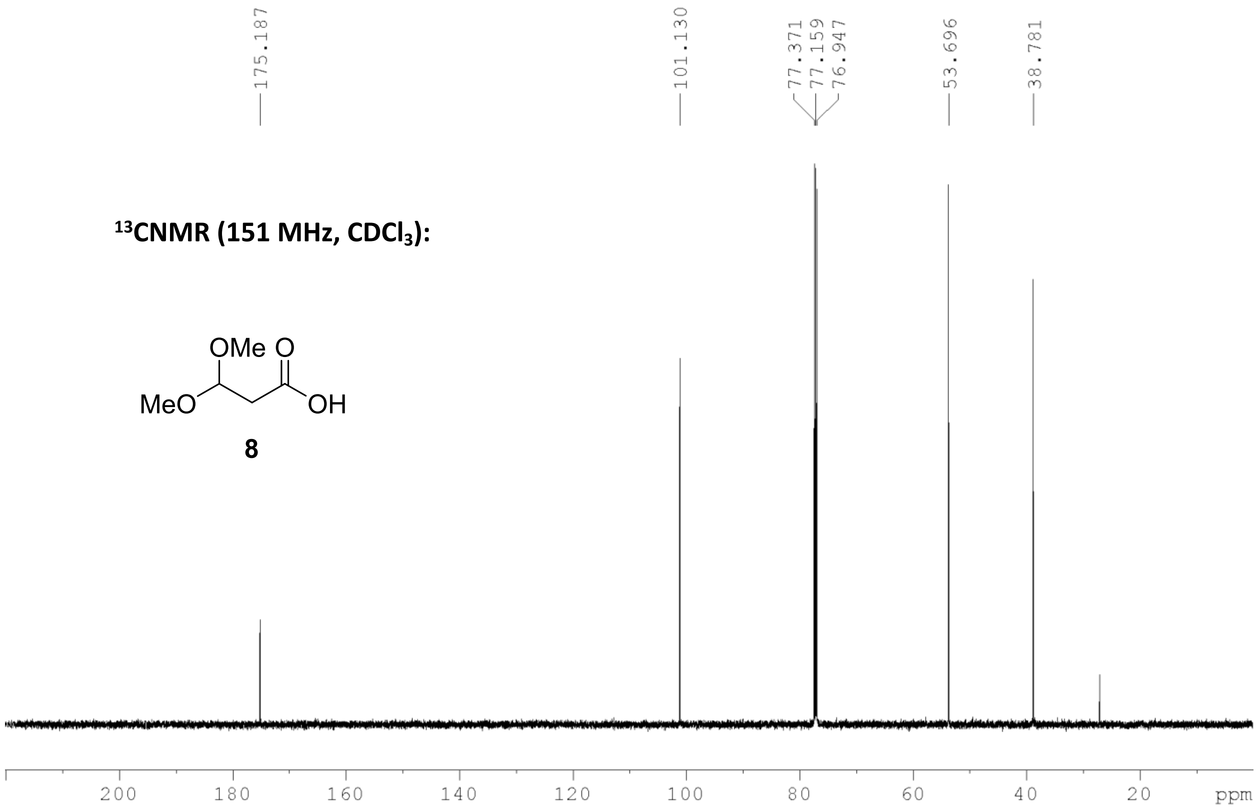

^{1}HNMR (600 MHz, d4-methanol):

6

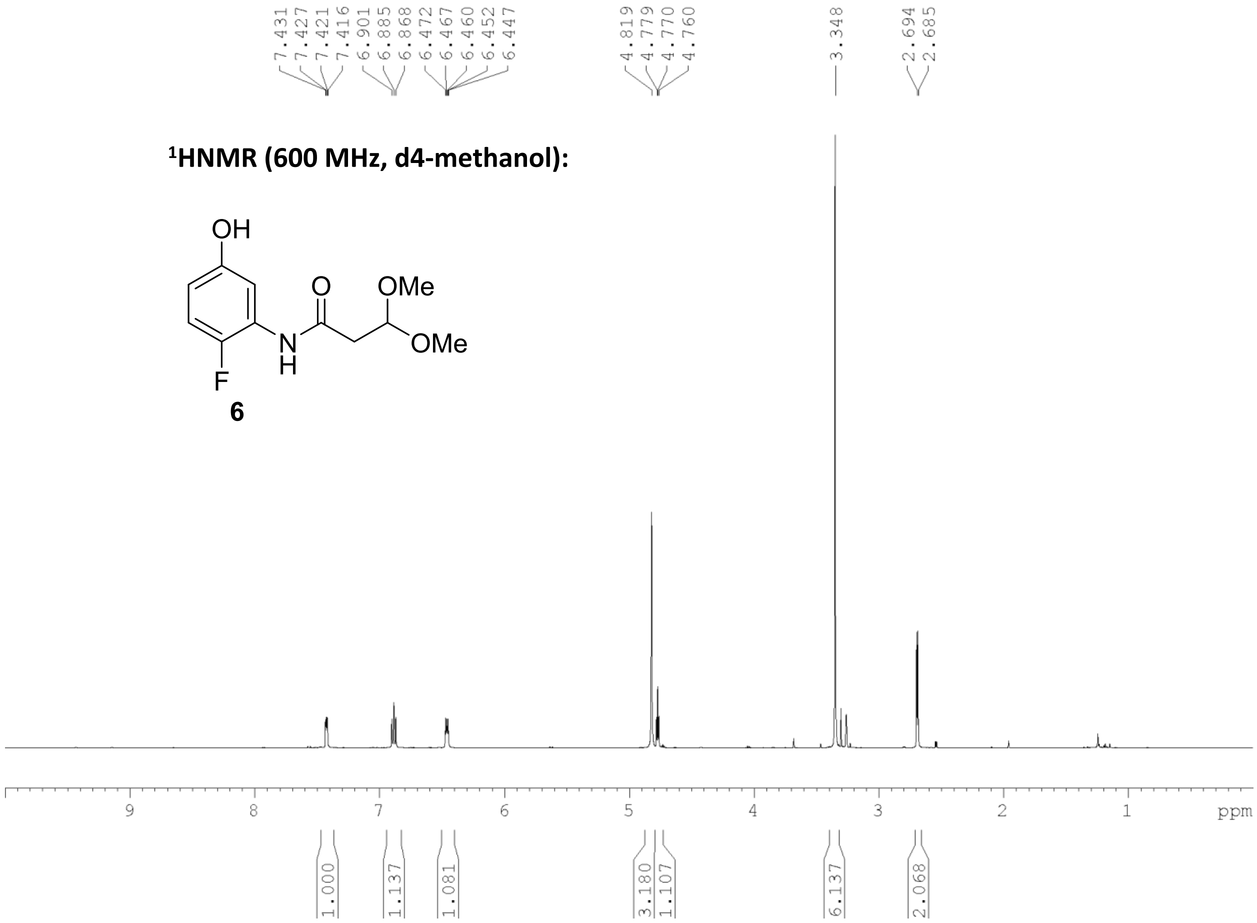

^{13}CNMR (151 MHz, d4-methanol):

6

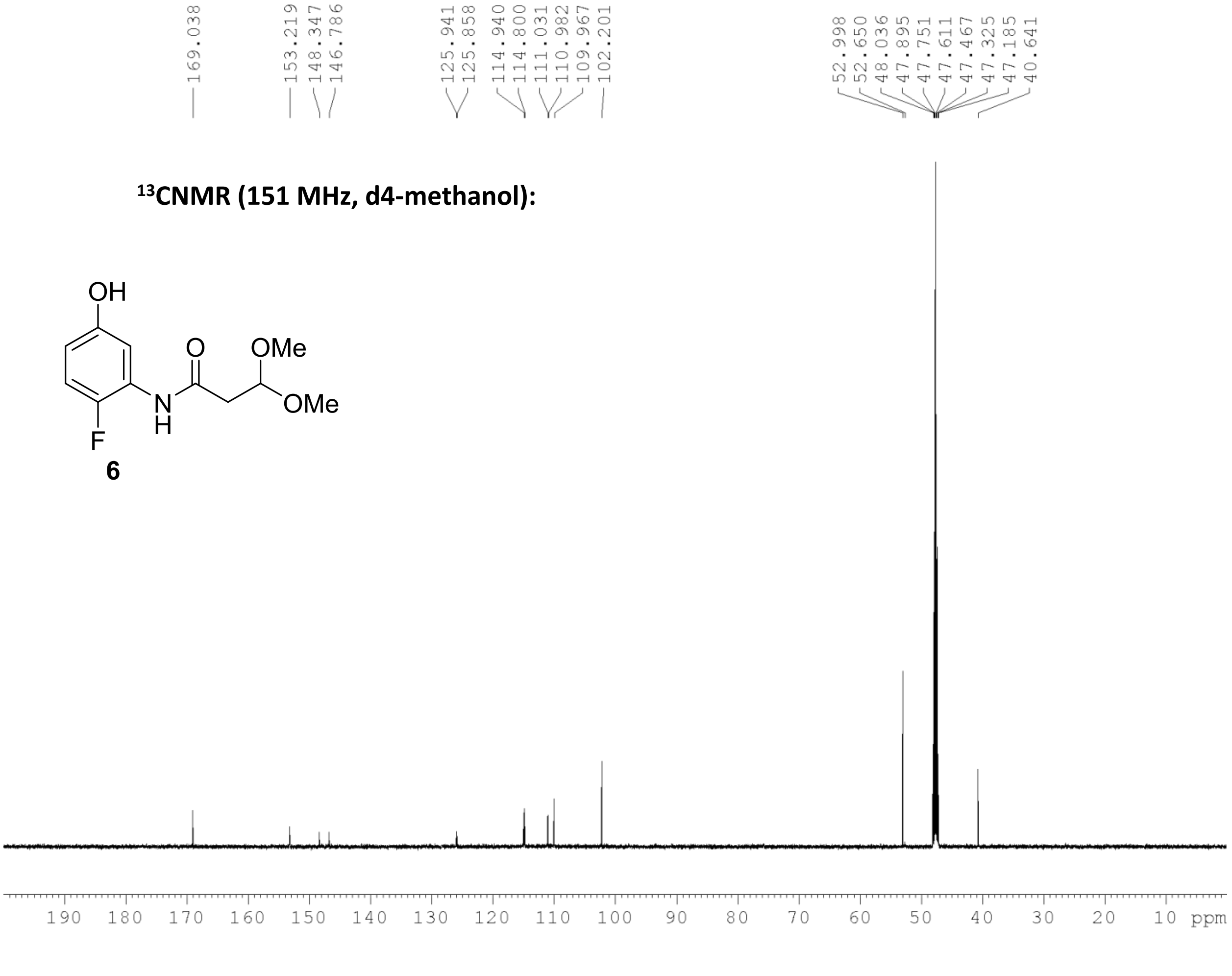

^{1}HNMR (600 MHz, d6-DMSO):

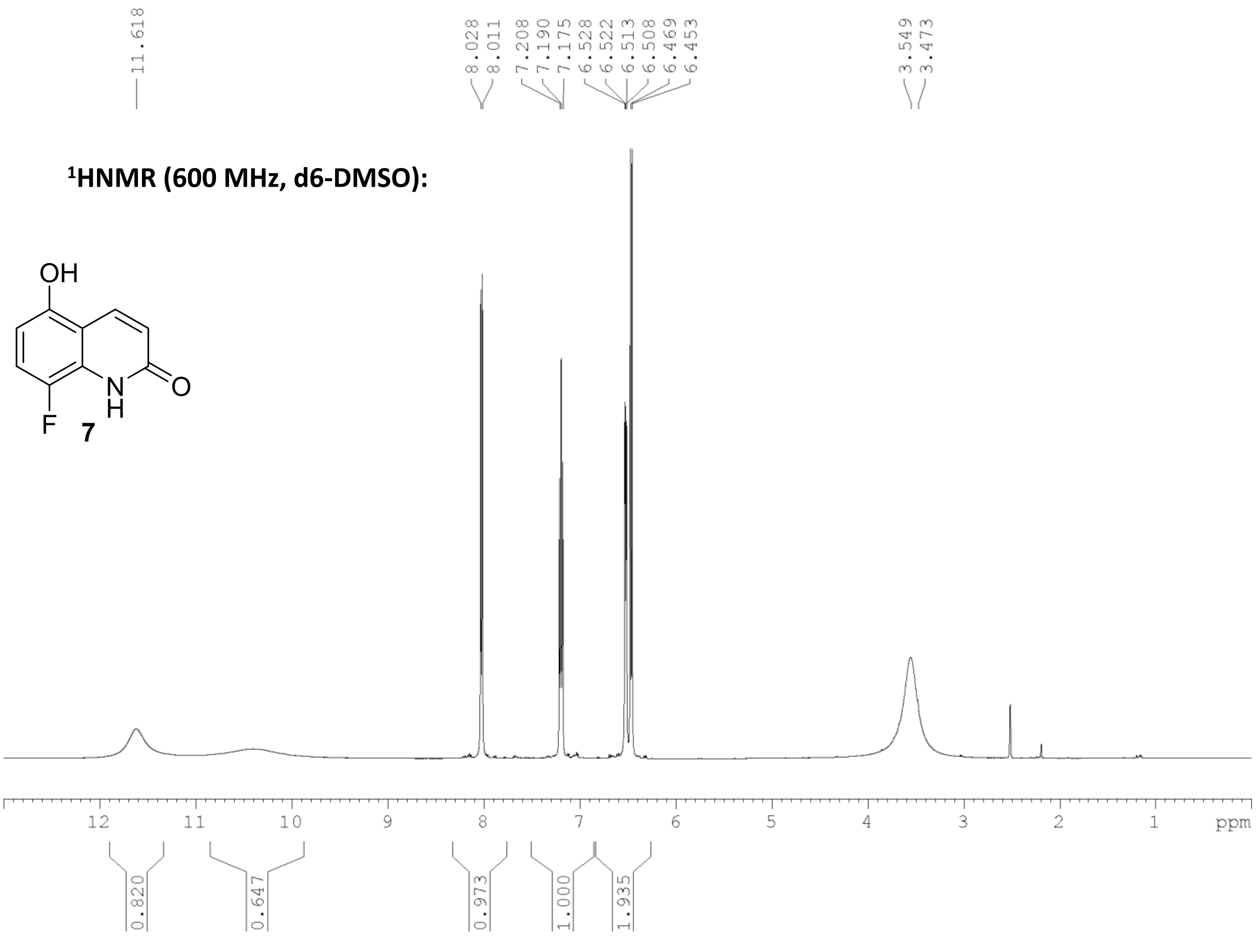

^{13}CNMR (151 MHz, d6-DMSO):

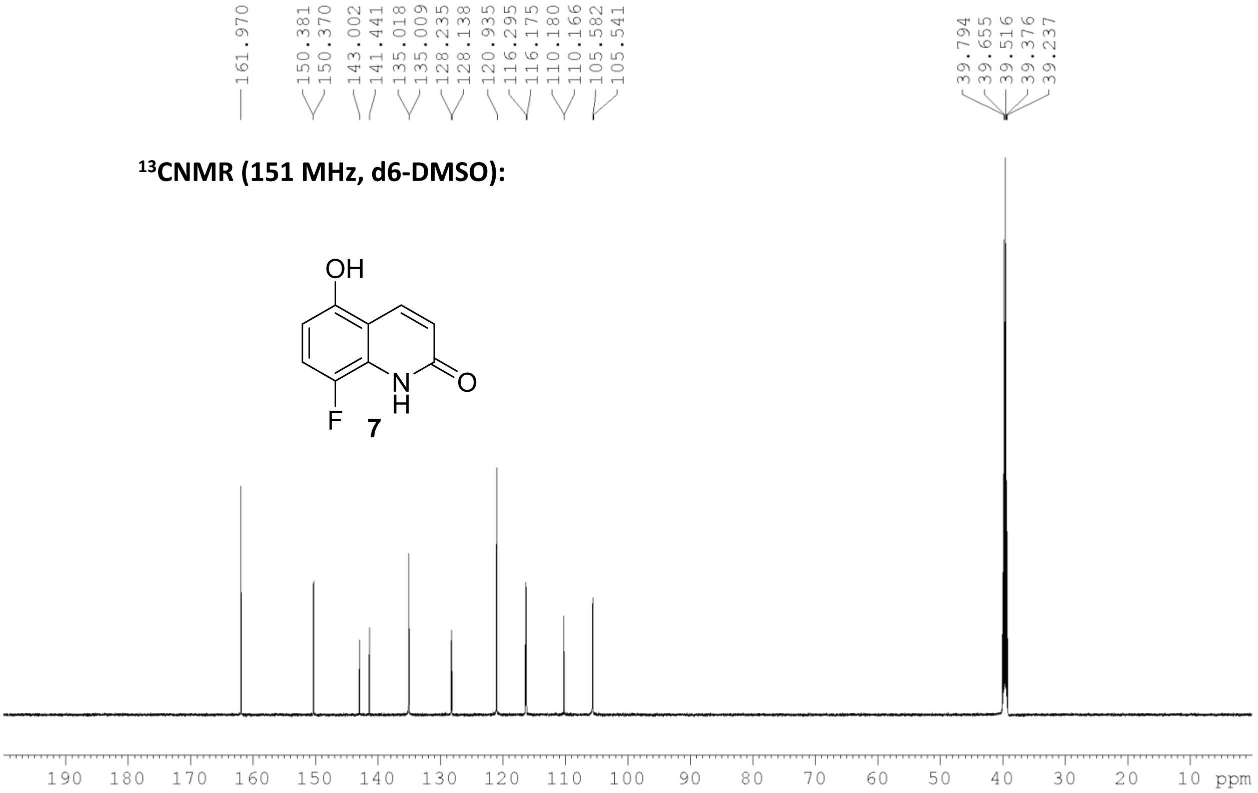

^{1}HNMR (600 MHz, d6-DMSO):

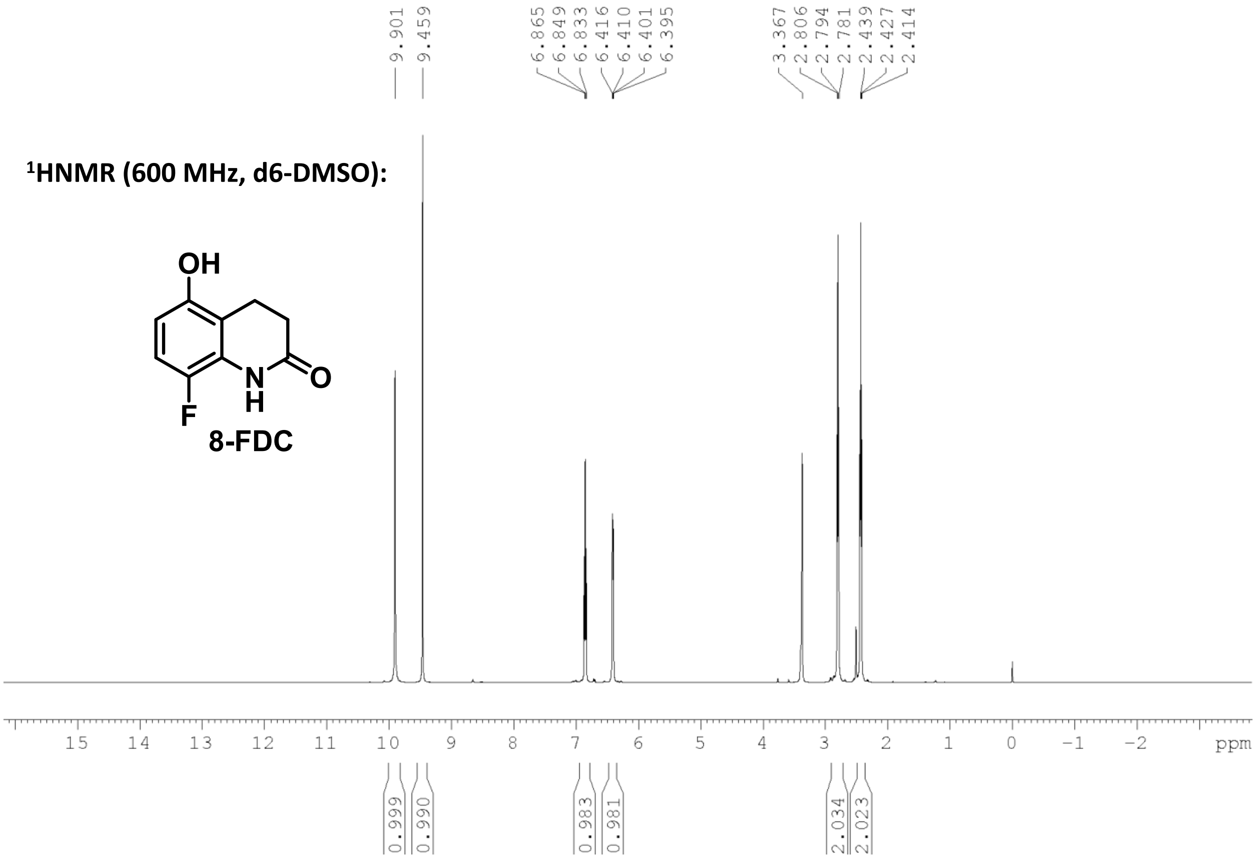

^{13}CNMR (151 MHz, d6-DMSO):

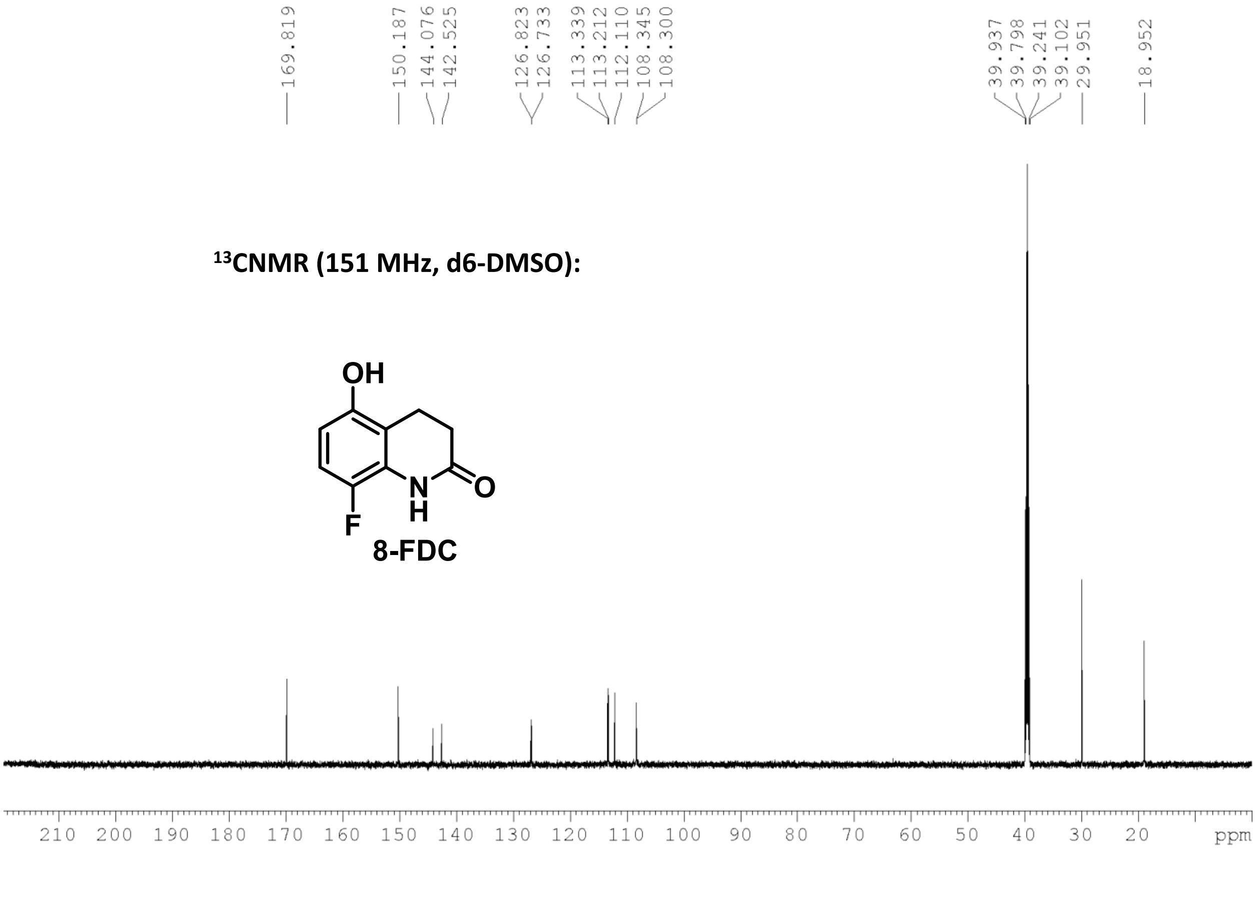

Supplement: Supplementary file 1 — ao1c06996_si_001.pdf [file ao1c06996_si_001.pdf]
